# Editorial: Antimicrobial Resistance Along the Food Chain: Are We What We Eat?

**DOI:** 10.3389/fmicb.2022.881882

**Published:** 2022-03-18

**Authors:** Aloysius Wong, Bojana Bogovic Matijasic, Joyce A. Ibana, Renee Lay Hong Lim

**Affiliations:** ^1^Department of Biology, College of Science and Technology, Wenzhou-Kean University, Wenzhou, China; ^2^Zhejiang Bioinformatics International Science and Technology Cooperation Center, Wenzhou, China; ^3^Wenzhou Municipal Key Lab for Applied Biomedical and Biopharmaceutical Informatics, Wenzhou, China; ^4^Department of Animal Science, Institute of Dairy Science and Probiotics, Biotechnical Faculty, University of Ljubljana, Ljubljana, Slovenia; ^5^Immunopharmacology Research Laboratory, Institute of Biology, College of Science, University of the Philippines, Quezon, Philippines; ^6^Department of Biotechnology, Faculty of Applied Sciences, UCSI University, Kuala Lumpur, Malaysia

**Keywords:** antibiotic resistance, resistome, horizontal gene transfer, agro-food chain, animal feed, zoonotic bacteria, whole genome sequence

Antimicrobial resistance (AR) is considered a “silent pandemic” that is responsible for more than 700,000 deaths per year; a figure that could rise to 10 million by 2050 if no action is taken (de Kraker et al., 2016; Mahoney et al., 2021). The use or rather misuse of antimicrobial drugs in hospitals has long been recognized as the main cause for the spread of antimicrobial resistant genes (ARGs) and this has become even more apparent during the SARS-CoV-2 pandemic where antimicrobial drugs were often prescribed unnecessarily to threat secondary infections (Knight et al., 2021; Russell et al., 2021; Wang et al., 2022).

In recent years, food production and agriculture among other anthropogenic activities, have exacerbated this problem (Van Boeckel et al., 2015; Wong et al., 2015; Caniça et al., 2019; Rozman et al., 2020; Schar et al., 2021). While the impact of clinical antimicrobial use and resistance has been well-documented, the contributions from other sources and how they fit into the overall prevalence of AR is less understood (Larsson and Flach, 2021). The underlying problem of AR is further complicated by the dynamic transmission of AR leading to the establishment of ARG reservoirs across various stages along the food chain (Imperial and Ibana, 2016; Hudson et al., 2017).

This Research Topic aims to offer a balanced overview of this global threat by gathering research focusing on AR along the food chain, from farm to fork. A total of 13 original research articles across China, India, Brazil, Malaysia, Spain, Iran, Egypt, and South Africa, have been published. Studies predicted that an overwhelming majority of people falling into poverty due to AR, will come from developing and underdeveloped countries, and our article collection reflects this geographical representation (Jit et al., 2020; Iskandar et al., 2021).

To demonstrate that farm animals are critical source for the dissemination of AR, Wu et al. evaluated the prevalence of *Salmonella* in a pig slaughtering house in China and found that the dehairing (66.66%) and splitting (57.14%) areas were the most contaminated with *Salmonella*. High frequency of resistance to tetracycline, ampicillin, chloramphenicol, and nalidixic acid, was also detected in the isolates. Bioinformatics analysis predicted a high dominance of *S. Typhimurium* ST19 among the isolates while several toxin encoding virulence factors were also identified.

In another study conducted on ducks in China, Wang et al. detected many ARGs from New Delhi metallo β-lactamase (NDM)-producing *Escherichia coli* isolates. Phenotypically, they show high frequency of resistance to trimethoprim-sulfamethoxazole, gentamicin, and fosfomycin. Conjugation experiments showed that the *bla*_*NDM*_-carrying plasmids is transferrable. Moreover, *bla*_*NDM*_ coexisted with other ARGs which suggests plausible transfer of ARGs among intestinal *E. coli* isolates of ducks.

A similar study conducted on shrimp aquaculture farms in India by Sivaraman et al. found that extended-spectrum β-lactamase (ESBL)-producing *E. coli* and *Klebsiella pneumoniae* isolates were resistant to cefotaxime, tetracycline, ciprofloxacin, and trimethoprim-sulfamethoxazole. At the molecular level, high prevalence of ARGs responsible for conferring resistance to β-lactamase (e.g., *bla*_*CTX*−*M*_), tetracyclines, sulfonamide and quinolone resistance, was detected.

In another study from Brazil, Cardozo et al. also detected high prevalence of *bla*_*CTX*−*M*_ in ESBL-producing *E. coli* and *K. pneumoniae* isolated from chicken, chicken meat, and human feces. The authors found high frequency of *bla*_*CTX*−*M*−15_ among the isolates although they are genetically diverse, which suggests that farm animals and their by-products, could be a source of transmission for ESBL-producing pathogens to humans.

In Malaysia, Zakaria et al. reported that *S. enteritidis* isolated from humans, poultry, and foods, was resistant to multiple drugs. ARGs responsible for resistance to aminoglycosides and tetracyclines were the most abundant, while other ARGs responsible for resistance to ampicillin, sulfonamide, and ciprofloxacin, were also detected. Similar to the findings of Cardozo et al., *S. enteritidis* isolates from the various sources share similar resistant genetic traits although they are from distinct lineages.

Zhang et al. also detected resistance to aminoglycosides in *Campylobacter* isolates from chicken and swine in China. The corresponding ARGs were determined, and conjugative experiments confirmed the transferability of aminoglycoside resistance among *C. jejuni* strains. The gene fragment responsible for the elevated resistance in recipient strains was also characterized.

Another study on aminoglycoside resistance by Lu et al. found that the prevalence rates of 16S rRNA methylation enzyme (*armA*)-harboring *Salmonella* strains were 1.1/1,000 and 8.7/1,000 in outpatient and food or environmental isolates, respectively. All *armA*-harboring *Salmonella* strains were resistant to multiple drugs. The *armA* gene was determined to be plasmid-borne and could be transferred to *E. coli* and *Acinetobacter baumannii*. Importantly, strains isolated from outpatients were genetically more identical to those from poultry than those from swine, thus inferring that poultry consumption is a credible source of infection.

In Egypt, Saber et al. examined methicillin- and vancomycin-resistant *Staphylococcus aureus* (MRSA and VRSA) isolated from ready-to-eat meat and food handlers. MRSA isolates were resistant to cefepime, penicillin, ampicillin-sulbactam, ciprofloxacin, nitrofurontoin, and gentamicin, while *VanA* and *VanB* resistant genes were detected in VRSA. Importantly, the isolates could form biofilm and they harbor several biofilm-forming genes, which suggest greater risk of colonization and dissemination.

In South Africa, Richter et al. detected multi-drug resistance in ESBL/AmpC β-lactamase(AmpC)-producing *E. coli, K. pneumoniae, Serratia fonticola*, and *S. enterica* isolates from spinach and irrigation water. Genes coffering resistance to different classes of antibiotics were detected with *bla*_*CTX*−*M*−15_ and bla_ACT_-types being the most dominant. *In silico* analysis predicted high similarities to human pathogens for all strains, implying contamination mediated by anthropogenic activities.

A large-scale resistome analysis of *Campylobacter spp*. genomes by Cobo-Díaz et al. found resistant determinants of β-lactams, tetracyclines, quinolones and aminoglycosides in their genomes many of which, are also frequently found together with genes conferring resistance to other antibiotics. The genomes of isolates from humans, food animals, and foods, contain higher frequency of ARGs responsible for resistance to tetracyclines and quinolones, possibly due to intense use of these drugs in veterinary and clinical settings.

Li et al. sequenced three multi-drug resistant *Listeria innocua* isolates from food. Unlike the listeriosis causing *L. monocytogenes, L. innocua* is not infectious but, the authors identified ARG islands in both chromosomes and plasmids. All isolates contain the pathogenicity island-4 (LIPI-4) and phylogenetic analysis revealed that they share common origins, thus suggesting transmission capability. This study advocates for surveillance on non-infectious strains to reveal the origins and concomitantly, track and contain the spread of AR in foods.

In hospitals, tigecycline and colistin are last-resort antibiotics used to threat infections. In this regard, Moghimi et al. investigated the mechanisms of tigecycline resistance and found that many non-susceptible *Klebsiella pneumoniae* isolates from human, food animals and/or laboratory selection experiments, are resistant to a combined treatment of tigecycline and colistin. All isolates from humans carried carbapenemase genes while high frequency of mutations in genes that led to increased expression of the AcrAB efflux pump, was detected. Since tigecycline is not used in animal farming, the detection of tigecycline resistance in animal isolates is thus, a clinical concern.

Environmentally sustainable approaches are being increasingly sought to eliminate ARGs from animal farming and in this regard, Peng et al. reported that treatment of animal manure with high heat effectively reduced ARGs introduced into soils. The authors showed that ARG abundance in chicken manure-treated soils was 1.41 times higher than that in mushroom residue-treated soils, but this difference was abrogated when heat-treated chicken manure-amended soils was used.

Contributions in this Research Topic incorporated a variety of approaches to collectively advance our understanding of AR across the different components beginning from farms and ending with the consumers. Through horizontal gene exchanges along the food chain, ARGs inevitably end up interacting with human microbiomes [EFSA Panel on Biological Hazards (BIOHAZ) et al., 2021]. Therefore, we hope this collection will encourage further research, and establish or expand AR surveillance in agriculture, environment, and food processing systems.

## Author Contributions

AW drafted the manuscript. All authors read, edited, and approved it for publication.

## Funding

AW would like to acknowledge funding from Wenzhou-Kean University under the Student Partnering with Faculty (SpF) research program (SpF2021002).

## Conflict of Interest

The authors declare that the research was conducted in the absence of any commercial or financial relationships that could be construed as a potential conflict of interest.

## Publisher's Note

All claims expressed in this article are solely those of the authors and do not necessarily represent those of their affiliated organizations, or those of the publisher, the editors and the reviewers. Any product that may be evaluated in this article, or claim that may be made by its manufacturer, is not guaranteed or endorsed by the publisher.

## References

[B1] Caniça M. Manageiro V. Abriouel H. Moran-Gilad J. Franz C. M. A. P. (2019). Antibiotic resistance in foodborne bacteria. Trends Food Sci. Technol. 84, 41–44. 10.1016/j.tifs.2018.08.001

[B2] de Kraker M. E. Stewardson A. J. Harbarth S. (2016). Will 10 Million people die a year due to antimicrobial resistance by 2050? PLoS Med. 13, e1002184. 10.1371/journal.pmed.1002184 PMC5127510 27898664

[B3] EFSA Panel on Biological Hazards (BIOHAZ) Koutsoumanis K. Allende A. Álvarez-Ordóñez A. Bolton D. Bover-Cid S.. (2021). Role played by the environment in the emergence and spread of antimicrobial resistance (AMR) through the food chain. EFSA J. 19, e06651. 10.2903/j.efsa.2021.6651 34178158 PMC8210462

[B4] Hudson J. A. Frewer L. J. Jones G. Brereton P. A. Wittingham M. J. Stewart G. (2017). The agri-food chain and antimicrobial resistance: a review. Trends Food Sci. Technol. 69, 131–147. 10.1016/j.tifs.2017.09.007

[B5] Imperial I. C. Ibana J. A. (2016). Addressing the antibiotic resistance problem with probiotics: reducing the risk of its double-edged sword effect. Front. Microbiol. 7, 1983. 10.3389/fmicb.2016.01983 28018315 PMC5156686

[B6] Iskandar K. Molinier L. Hallit S. Sartelli M. Hardcastle T. C. Haque M.. (2021). Surveillance of antimicrobial resistance in low- and middle-income countries: a scattered picture. Antimicrob. Resist. Infect. Control 10, 63. 10.1186/s13756-021-00931-w 33789754 PMC8011122

[B7] Jit M. Ng D. H. L. Luangasanatip N. Sandmann F. Atkins K. E. Robotham J. V.. (2020). Quantifying the economic cost of antibiotic resistance and the impact of related interventions: rapid methodological review, conceptual framework and recommendations for future studies. BMC Med. 18, 38. 10.1186/s12916-020-1507-2 32138748 PMC7059710

[B8] Knight G. M. Glover R. E. McQuaid C. F. Olaru I. D. Gallandat K. Leclerc Q. J.. (2021). Antimicrobial resistance and COVID-19: Intersections and implications. Elife 10, e64139. 10.7554/eLife.64139 33588991 PMC7886324

[B9] Larsson D. G. J. Flach C. F. (2021). Antibiotic resistance in the environment. Nat. Rev. Microbiol. 1–13. 10.1038/s41579-021-00649-x [Epub ahead of print]. PMC8567979 34737424

[B10] Mahoney A. R. Safaee M. M. Wuest W. M. Furst A. L. (2021). The silent pandemic: Emergent antibiotic resistances following the global response to SARS-CoV-2. iScience 24, 102304. 10.1016/j.isci.2021.102304 33748695 PMC7955580

[B11] Rozman V. Mohar Lorbeg P. Accetto T. Bogovič Matijašić B. (2020). Characterization of antimicrobial resistance in lactobacilli and bifidobacteria used as probiotics or starter cultures based on integration of phenotypic and in silico data. Int. J. Food Microbiol. 314, 108388. 10.1016/j.ijfoodmicro.2019.108388 31707173

[B12] Russell C. D. Fairfield C. J. Drake T. M. Turtle L. Seaton R. A. Wootton D. G.. (2021). Co-infections, secondary infections, and antimicrobial use in patients hospitalised with COVID-19 during the first pandemic wave from the ISARIC WHO CCP-UK study: a multicentre, prospective cohort study. Lancet Microbe. 2, e354–e365. 10.2139/ssrn.3786694 34100002 PMC8172149

[B13] Schar D. Zhao C. Wang Y. Larsson D. G. J. Gilbert M. Van Boeckel T. P. (2021). Twenty-year trends in antimicrobial resistance from aquaculture and fisheries in Asia. Nat. Commun. 12, 5384. 10.1038/s41467-021-25655-8 34508079 PMC8433129

[B14] Van Boeckel T. P. Brower C. Gilbert M. Grenfell B. T. Levin S. A. Robinson T. P.. (2015). Global trends in antimicrobial use in food animals. Proc. Natl. Acad. Sci. U. S. A. 112, 5649–5654. 10.1073/pnas.1503141112 25792457 PMC4426470

[B15] Wang Y. Dong J. Wang J. Chi W. Zhou W. Tian Q.. (2022). Assessing the drug resistance profiles of oral probiotic lozenges. J. Oral Microbiol. 14, 2019992. 10.1080/20002297.2021.2019992 35024089 PMC8745366

[B16] Wong A. Ngu D. Y. Dan L. A. Ooi A. Lim R. L. (2015). Detection of antibiotic resistance in probiotics of dietary supplements. Nutr. J. 14, 95. 10.1186/s12937-015-0084-2 26370532 PMC4568587

